# Acupuncture Alleviates Neuroinflammation in Chronic Migraine by Modulating *Lactobacillus* and Its Metabolite Pathways

**DOI:** 10.1155/prm/5189419

**Published:** 2026-06-23

**Authors:** Shiqi Sun, Longyao Xu, Yuyan Wang, Shuangyuan Hu, Mingsheng Sun, Jing Yuan, Ling Zhao

**Affiliations:** ^1^ Acupuncture and Tuina School, Chengdu University of Traditional Chinese Medicine, Chengdu, Sichuan, China, cdutcm.edu.cn; ^2^ Key Laboratory of Acupuncture and Moxibustion in Prevention and Treatment of Geriatric Diseases (Chengdu University of Traditional Chinese Medicine), Ministry of Education, Chengdu, Sichuan, China, meb.gov.tr

**Keywords:** chronic migraine, gut–brain axis, gut microbiota, *Lactobacillus*, metabolomics

## Abstract

**Background:**

Chronic migraine (CM) is a highly prevalent and disabling neurological disorder lacking universally effective treatments. Acupuncture has shown significant clinical efficacy, yet its precise mechanisms remain unclear. This study aimed to evaluate the therapeutic effects and underlying mechanisms of acupuncture in CM, integrating gut microbiota and metabolomic analyses.

**Materials and Methods:**

Forty‐two Sprague–Dawley rats were randomly assigned to seven groups: control (Con), CM model (Mod), acupuncture (Acu), model + probiotics (Mod + Pro), model + antibiotics (Mod + Anti), acupuncture + probiotics (Acu + Pro), and acupuncture + antibiotics (Acu + Anti). CM was induced via subcutaneous nitroglycerin injection. Acupuncture was performed for nine days at bilateral Shuaigu (GB8) and Yanglingquan (GB34) points (20 min/day). Probiotics were administered by oral gavage of a mixed *Lactobacillus* preparation for 9 days; antibiotics were given as an oral cocktail for 2 weeks premodeling. Pain sensitivity and central inflammation were assessed by behavioral tests and ELISA. Gut microbiota and metabolites were profiled using 16S rDNA sequencing and metabolomics.

**Results:**

Acupuncture alleviated pain hypersensitivity and central inflammation, reversing CM‐induced gut dysbiosis, with marked effects on Akkermansia muciniphila and *Lactobacillus*. Metabolomics identified multiple altered metabolites, with strong correlations between *Limosilactobacillus* and unclassified_Lactobacillaceae and cis‐5‐dodecenoic acid and dihydrolipoamide. Network analysis revealed *Limosilactobacillus* as a core node, suggesting modulation of gut–brain axis signaling via specific metabolic pathways. Exogenous *Lactobacillus* markedly alleviated hyperalgesia and inflammation in model rats, with further analgesic and anti‐inflammatory potentiation when combined with acupuncture.

**Conclusion:**

Acupuncture may exert antimigraine effects by modulating the *Lactobacillus*–metabolite–inflammation axis, restoring gut homeostasis, and alleviating pain and neuroinflammation. Probiotic supplementation further supports the role of gut microbiota in mediating acupuncture’s benefits, offering insight for mechanistic studies and clinical translation.

## 1. Introduction

Chronic migraine (CM) is a prevalent neurological disorder characterized by high incidence, substantial disability, and considerable socioeconomic burden [1, 2]. Its pathophysiology is multifactorial, involving aberrant central sensitization, neurogenic inflammation, neurovascular dysfunction, and neurotransmitter imbalance [3, 4]. Current therapeutic strategies primarily rely on pharmacological prophylaxis and acute‐phase management; however, treatment efficacy varies markedly among individuals, and long‐term use is often limited by adverse effects or drug tolerance, resulting in suboptimal clinical control [5, 6]. Therefore, the development of effective interventions that are both safe and capable of holistic regulation holds significant importance for the treatment of CM and the investigation of its underlying mechanisms.

In recent years, the microbiota–gut–brain axis (MGBA), a bidirectional regulatory network linking the gut microbial ecosystem with the central nervous system, has emerged as a critical factor in various neurological disorders. Accumulating evidence indicates that CM is frequently accompanied by gastrointestinal dysfunction and gut dysbiosis, with the resulting microecological disturbances contributing to the initiation or exacerbation of neuroinflammation and central sensitization via inflammatory mediators, neurotransmitters, and microbially derived metabolites [7, 8]. Moreover, gut barrier impairment—leading to lipopolysaccharide (LPS) translocation and short‐chain fatty acid (SCFA) imbalance—has been implicated in the persistence of chronic pain [9–11]. These metabolites, through immune and neural signaling pathways, can activate microglia, induce neuroinflammatory responses, and heighten pain sensitization [12, 13]. Thus, restoring gut homeostasis and re‐establishing the microbiota–metabolite network may offer novel avenues for mitigating central inflammation and pain in CM.

As an integral component of traditional Chinese medicine, acupuncture is widely applied for the management of various pain disorders owing to its robust analgesic effects and systemic regulatory capacity [14–16]. Multiple clinical and experimental studies have demonstrated the significant efficacy of acupuncture in CM management [17–19]. Notably, studies in chronic pain and central inflammatory disorders have suggested that acupuncture may modulate gut motility, permeability, and microbial ecology through activation of the vagus nerve, regulation of the autonomic nervous system, and modulation of the hypothalamic–pituitary–adrenal (HPA) axis, thereby enabling remote regulation of the MGBA [20–22]. However, studies in the field of pain modulation remain relatively limited, and systematic investigations elucidating whether acupuncture can intervene in the pathogenesis of CM through the complex pathway of “gut microbiota–metabolites–central neuroinflammation” are still lacking.

Based on this rationale, the present study employed a CM rat model in combination with 16S rRNA sequencing and untargeted metabolomics to comprehensively investigate the therapeutic characteristics and potential mechanisms of acupuncture from the perspective of the interlinked “microbiota–metabolite–neuroinflammation” axis. The findings aim to provide a solid mechanistic foundation for acupuncture in CM treatment and to extend its application in the fields of microecology and neuroimmune regulation.

## 2. Materials and Methods

### 2.1. Drugs and Reagents

Nitroglycerin injection (Approval No. H11020289) was purchased from Beijing Yimin Pharmaceutical Co., Ltd. (Beijing, China). The mixed *Lactobacillus* preparation consisted of *Lactobacillus plantarum*, *Lactobacillus paracasei*, *Lactobacillus* acidophilus, and *Lactobacillus delbrueckii subsp. bulgaricus* and was obtained from Beijing Beina Chuanglian Biotechnology Research Institute (Beijing, China). Enzyme‐linked immunosorbent assay (ELISA) kits were supplied by Shanghai Zhuocai Biotechnology Co., Ltd. (Shanghai, China).

### 2.2. Animals and Group Allocation

Healthy male Sprague–Dawley (SD) rats (200 ± 20 g, SPF grade) were obtained from Chengdu Enswire Experimental Animal Co., Ltd. (Chengdu, China). All animals were housed under standard laboratory conditions (temperature: 24°C ± 2°C; humidity: 40%–70%; 12 h light/dark cycle) with ad libitum access to food and water. Rats were acclimatized to the housing environment for 7 days prior to experimentation. To avoid cross‐interference of gut microbiota, animals from different experimental groups were housed separately.

A total of 42 rats were randomly allocated using a random number table into seven groups (*n* = 6 per group): control (Con), CM model (Mod), acupuncture (Acu), model + probiotics (Mod + Pro), model + antibiotics (Mod + Anti), acupuncture + probiotics (Acu + Pro), and acupuncture + antibiotics (Acu + Anti). All experimental procedures were approved by the Experimental Animal Ethics Committee of Chengdu University of Traditional Chinese Medicine (Approval No. 2019‐23) and were conducted in accordance with the Guidelines for the Care and Use of Laboratory Animals of the Chinese Pharmacological Society.

### 2.3. Model Establishment and Intervention

The CM model was induced by subcutaneous injection of nitroglycerin (NTG, 10 mg/kg) once every other day for nine consecutive days. During the intervention phase, rats in the acupuncture group received daily acupuncture treatment at bilateral Shuaigu (GB8; located at the midpoint between the auricular apex and the parietal tubercle) and Yanglingquan (GB34; located inferior to the fibular head on the lateral aspect of the knee) acupoints. A single‐use sterile acupuncture needle (0.25 ∗ 25 mm, Suzhou Huatuo Medical Equipment Co., Ltd., Suzhou) was used for each session. Acupuncture was performed bilaterally at both acupoints and nonacupoint sites, with a needle insertion depth of approximately 5‐6 mm. Acupuncture treatment began on the first day of modeling and was performed once per day for 20 min per session, lasting for a total of 9 days. The blank and model groups were similarly restrained for 20 min daily, but no acupuncture treatment was applied.

**FIGURE 1:**
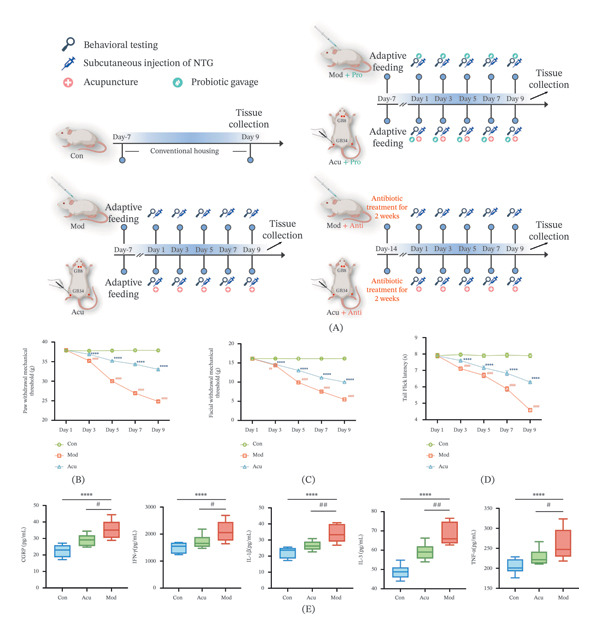
Experimental workflow and behavioral/ELISA results. (A) Schematic diagram of the experimental design; (B) comparison of PWMT among Con, Mod, and Acu groups during the intervention period; (C) comparison of FWMT among groups; (D) comparison of TFL among groups; and (E) ELISA results showing brain inflammatory cytokine levels among the Con, Mod, and Acu groups (box plots). *n* = 6; vs. con: *p* < 0.05, ^∗^ *p* < 0.01, ^∗∗^ *p* < 0.001, ^∗∗∗^ *p* < 0.0001; vs. mod: ^#^ *p* < 0.05, ^##^ *p* < 0.01, ^###^ *p* < 0.001, ^####^ *p* < 0.0001.

For depletion of gut microbiota, rats in the Anti group were given ad libitum access to an antibiotic cocktail dissolved in sterile drinking water, consisting of ampicillin (1.0 g/L), neomycin (1.0 g/L), metronidazole (1.0 g/L), and vancomycin (0.5 g/L). The antibiotic solution was replaced every 1‐2 days, and administration commenced 2 weeks prior to model induction to maintain drug efficacy and stability. To ensure adequate antibiotic intake, in addition to measuring and recording the animals’ water consumption daily, the concentration of antibiotics in the drinking water is adjusted every 5 days based on the animals’ water consumption and body weight, ensuring consistent antibiotic intake. Rats in the Pro group received mixed *Lactobacillus* supplementation via oral gavage (2.7 × 10^9^ CFU/rat/day) during the modeling period for a total of 9 days. A schematic of the experimental design is shown in Figure 1A.

### 2.4. Pain‐Related Behavioral Assessments

All pain‐related behavioral tests were conducted in a blinded manner and included the paw withdrawal mechanical threshold (PWMT), facial withdrawal mechanical threshold (FWMT), and tail‐flick latency (TFL). PWMT and FMWT were assessed using Von Frey filaments to stimulate the central plantar surface and periorbital region of the rats, respectively, to evaluate changes in mechanical sensitivity. TFL was determined by applying a thermal stimulus to the tail and recording the latency from stimulus onset to tail withdrawal, reflecting thermal nociceptive sensitivity. Behavioral assessments were performed on Days 1, 3, 5, 7, and 9 after model induction to dynamically monitor the progression of pain hypersensitivity and the therapeutic effects in each group.

### 2.5. Sample Collection

For microbiota and metabolomics analyses, at least five fresh fecal pellets were collected directly from the rectum of each rat, placed into prechilled sterile centrifuge tubes, and immediately stored at −80°C. Under anesthesia with 3% sodium pentobarbital, rats underwent cardiac perfusion, after which brain tissues were rapidly dissected. The spinal trigeminal nucleus caudalis (Sp5C) region was quickly isolated, snap‐frozen in liquid nitrogen, and stored at −80°C until further analysis.

### 2.6. ELISA

The concentrations of calcitonin gene‐related peptide (CGRP), interferon‐gamma (IFN‐γ), interleukin‐1 beta (IL‐1β), interleukin‐3 (IL‐3), and tumor necrosis factor‐alpha (TNF‐α) in brain tissue were measured using commercially available ELISA kits according to the manufacturer’s protocols.

### 2.7. 16S Ribosomal RNA (rRNA) Gene Sequencing

Total DNA was extracted from fecal samples, and the target regions of the 16S rRNA gene were amplified using specific primers. Amplicons were pooled at equimolar concentrations and subjected to high‐throughput sequencing. Raw sequencing data underwent quality control (QC), including sequence filtering, chimera removal, and operational taxonomic unit clustering. Amplicon sequence variant analysis was then performed to identify unique variants, followed by taxonomic annotation using reference databases. Alpha (α) and beta (β) diversity metrics were calculated using the QIIME2 platform to evaluate intra‐ and intersample microbial diversity. Linear discriminant analysis effect size (LEfSe) was employed to identify taxa with significantly different abundances (LDA ≥ 4.0, *p* ≤ 0.05), while PICRUSt2 was used for functional prediction and annotation of Kyoto Encyclopedia of Genes and Genomes (KEGG) metabolic pathways, as well as for differential pathway analysis.

### 2.8. Liquid Chromatography–Tandem Mass Spectrometry (LC–MS/MS) Analysis

Fecal metabolomic profiling was performed using a Waters Acquity I‐Class PLUS ultra‐performance liquid chromatography (UHPLC) system coupled with a Xevo G2‐XS QToF high‐resolution mass spectrometer. Data acquisition was conducted in both positive and negative ionization modes, using MSe data‐independent acquisition (DIA). Raw data were processed with Progenesis QI software for peak extraction and metabolite identification. An orthogonal partial least squares‐discriminant analysis (OPLS‐DA) model and its permutation test were employed to evaluate the robustness and discriminatory power of the model. Differential metabolites were selected based on the following criteria: variable importance in projection (VIP) > 1.0, *p* < 0.05, and fold change (FC) ≥ 1.2 or ≤ 0.83. The identified metabolites were further subjected to pathway enrichment analysis using the KEGG database to evaluate their biological functions and potential mechanisms. In addition, log_2_FC values were calculated for the differential metabolites, and the top 10 metabolites ranked by the absolute value of log_2_FC were selected to generate radar plots illustrating their variation trends.

### 2.9. Microbiota–Metabolite Correlation Analysis

Microbe–metabolite pairs with an absolute correlation coefficient (|CC|) > 0.8 and *P* (CCP) < 0.05 were first identified to generate a correlation heatmap between differential metabolites and bacterial genera. Pairs meeting *p* < 0.05 were then further filtered, and the occurrence frequency of each metabolite and bacterial genus was calculated. The top 30 most frequently occurring metabolites and genera were selected for further analysis. To ensure that critical associations were not overlooked, all microbe–metabolite pairs containing at least one CC ranked within the top 30 in absolute value were retained. Finally, a chord diagram was constructed to visualize the correlations between metabolite modules and bacterial genera.

### 2.10. Statistical Analysis

All statistical analyses were performed using IBM SPSS Statistics Version 25.0, and figures were generated with GraphPad Prism version 8.0. Data are presented as mean ± standard deviation (mean ± SD). Comparisons between two groups were conducted using a two‐tailed unpaired Student’s *t*‐test, while comparisons among three or more groups were performed using one‐way or two‐way analysis of variance (ANOVA), depending on the experimental design. Unless otherwise specified, statistical significance was defined as: ^∗^ *p* < 0.05, ^∗∗^ *p* < 0.01, ^∗∗∗^ *p* < 0.001 and ^∗∗∗∗^ *p* < 0.0001.

## 3. Results

### 3.1. Acupuncture Significantly Alleviated NTG‐Induced Pain Hypersensitivity and Central Neuroinflammation in Rats

To validate the establishment of the CM model and evaluate the therapeutic effects of acupuncture on NTG‐induced pain hypersensitivity and central neuroinflammation, pain‐related behavioral parameters—including PWMT, FWMT, and TFL—were assessed on Days 1, 3, 5, 7, and 9 after model induction.

Compared with the Con, the Mod exhibited a sustained and significant decrease in pain thresholds over the 9‐day modeling period. Marked differences in mechanical and thermal responses were observed from Day 3 onward (*p* < 0.0001), confirming that the CM model successfully induced a stable pain hypersensitivity state (Figure 1B–D; Tables S1–S3). The Acu group produced a significant analgesic effect in PWMT and FWMT from Day 3 (*p* < 0.0001), with thresholds continuing to improve as the intervention progressed. Similarly, TFL was significantly prolonged from day 3 (*p* < 0.05), with more pronounced differences by Day 9 (*p* < 0.0001), indicating that acupuncture exerted a stable and sustained effect in alleviating central sensitization.

To further explore the anti‐inflammatory mechanism of acupuncture, ELISAs were performed to quantify inflammation‐related cytokines in the Sp5C region. Compared with the Con group, the Mod group exhibited markedly elevated expression of CGRP, IFN‐γ, IL‐1β, IL‐3, and TNF‐α (*p* < 0.0001), indicating that NTG induction triggered a robust central inflammatory response. Acupuncture significantly reduced the expression levels of these inflammatory mediators, with CGRP, IFN‐γ, IL‐3, and TNF‐α showing significant decreases (*p* < 0.05) and IL‐1β exhibiting a more pronounced reduction (*p* < 0.01) compared with the Mod group (Figure 1E). Collectively, these findings demonstrate that acupuncture effectively attenuates NTG‐induced central neuroinflammation and has the potential to alleviate pain hypersensitivity associated with CM.

### 3.2. Acupuncture Reverses Gut Microbiota Dysbiosis Induced by CM

To investigate whether the therapeutic effects of acupuncture on CM are associated with modulation of the gut microbiota, 16S rRNA gene sequencing was performed on fecal samples from the Con, Mod, and Acu groups. Multiple α‐diversity indices, including Chao1, Shannon, Simpson, and ACE, were calculated to assess species richness and diversity among groups (Figures S1A–S1D). The results showed no significant differences in these indices across groups, suggesting that neither CM induction nor acupuncture intervention markedly altered the overall species count or evenness of the gut microbiota.

**FIGURE 2:**
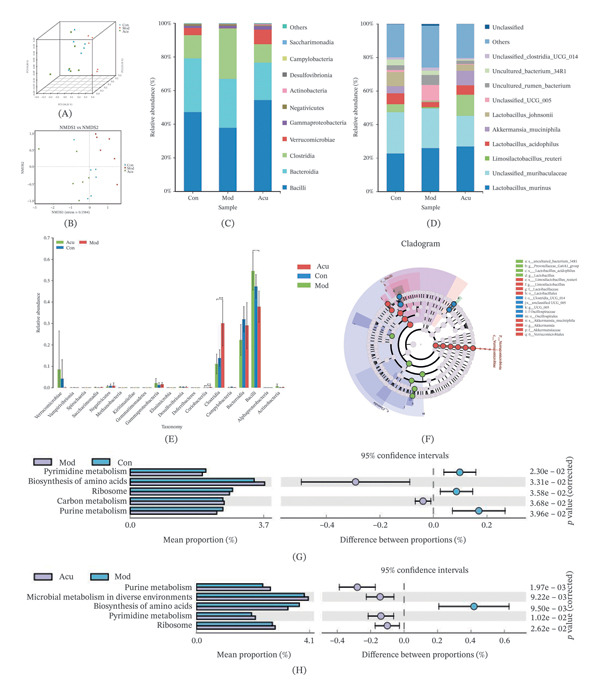
Comparative analysis of gut microbiota among experimental groups. (A) Principal coordinates analysis (PCoA) based on species‐level composition; (B) nonmetric multidimensional scaling (NMDS) plot showing β‐diversity patterns; (C) relative abundance of gut microbiota at the class level; (D) relative abundance of gut microbiota at the species level; (E) one‐way ANOVA analysis of taxa at the class level; (F) linear discriminant analysis effect size (LEfSe) cladogram highlighting significantly different microbial lineages among groups; (G) predicted KEGG metabolic pathways (level 3) between con and mod groups based on PICRUSt2 analysis; and (H) predicted KEGG metabolic pathways (level 3) between con and Acu groups based on PICRUSt2 analysis. ^∗^ *p* < 0.05, ^∗∗^ *p* < 0.01.

To further characterize differences in microbial community composition, principal coordinates analysis (PCoA) and nonmetric multidimensional scaling (NMDS) were employed for β‐diversity visualization. This combined approach allows simultaneous evaluation of absolute differences in microbial communities and their ordination relationships, providing a more comprehensive and robust assessment of intergroup microecological variation. As shown in (Figure 2A,B), the Mod group exhibited clear separation from the Con group at the species level, indicating that CM induced significant gut microbiota dysbiosis. Notably, the Acu group, following acupuncture intervention, displayed a microbial community structure that shifted toward that of the Con group, suggesting a trend toward restoration of normal microecological status. These findings imply that acupuncture may exert its antimigraine effects, at least in part, through the modulation of gut microbial community composition.

In the overall compositional analysis of the gut microbiota, taxonomic bar plots were generated to illustrate the dominant taxa at different classification levels in each group. At the class level, Bacilli, Bacteroidia, Clostridia, Verrucomicrobiae, and Gammaproteobacteria represented the five most abundant taxa (Figure 2C). At the species level, *Lactobacillus* murinus, unclassified_Muribaculaceae, *Limosilactobacillus reuteri*, *Lactobacillus* acidophilus, Akkermansia muciniphila, and *Lactobacillus johnsonii* were predominant (Figure 2D). These bacterial taxa are widely recognized for their critical roles in gut–brain axis signaling and the maintenance of metabolic homeostasis.

Based on the overall microbial distribution patterns, ANOVA was performed at the class level (Figure 2E, Table S4) to identify core taxa exhibiting significant dysregulation in the CM model that could be modulated by acupuncture. The results revealed that *Clostridia* was significantly decreased in the Mod group compared with the Con group (Con vs. Mod, *p* < 0.01), a change markedly reversed by acupuncture (Acu vs. Mod, *p* < 0.001). Bacilli were significantly enriched in the Acu group (Acu vs. Mod, *p* < 0.01), whereas *Coriobacteriia* showed significant alterations in the Mod group (Con vs. Mod, *p* < 0.01) and was partially restored following acupuncture treatment (Acu vs. Mod, *p* < 0.05). These differential taxa are potentially linked to key functional pathways such as SCFA synthesis, lipid metabolism, and the transport of aromatic compounds.

To pinpoint specific microbial biomarkers modulated by acupuncture at higher taxonomic resolution, LEfSe analysis was conducted (Figure 2F). In the Con group, dominant taxa were mainly clustered within the lineage s_Lactobacillus acidophilus ⟶ g_Lactobacillus. In contrast, the Mod group displayed a scattered distribution without well‐defined dominant lineages, reflecting disrupted gut microecological architecture and reduced functional stability. Notably, the Acu group exhibited relatively complete and systematic phylogenetic lineages, including s_Akkermansia muciniphila ⟶ g_Akkermansia ⟶ f_Akkermansiaceae ⟶ o_Verrucomicrobiales and s_Limosilactobacillus reuteri ⟶ g_Limosilactobacillus ⟶ f_Lactobacillaceae ⟶ o_Lactobacillales, suggesting that acupuncture preferentially enriches mucin‐degrading bacteria and lactic acid bacteria—key taxa with recognized roles in metabolism and barrier protection—during the restoration of gut homeostasis.

To explore potential functional shifts underlying the observed community changes, functional predictions were performed using PICRUSt2. The analysis revealed significant differences between the Con and Mod groups in core KEGG pathways such as pyrimidine metabolism, biosynthesis of amino acids, ribosome, carbon metabolism, and purine metabolism (Figure 2G), indicating that CM modeling not only reshaped microbial composition but also potentially restructured fundamental metabolic networks associated with nucleic acid and protein biosynthesis, as well as energy and carbon flow. Correspondingly, differences between the Mod and Acu groups were also concentrated in purine/pyrimidine metabolism, ribosome, amino acid biosynthesis, and carbon metabolism, with additional enrichment in microbial metabolism in diverse environments (Figure 2H), suggesting that acupuncture may facilitate functional recovery by reprogramming the material and energy metabolic potential of the gut microbiota.

### 3.3. Acupuncture Reverses Metabolic Dysregulation in CM Rats

Having confirmed that acupuncture modulates the composition and abundance of gut microbiota, we next investigated whether this intervention also alters the metabolic profile in CM rats. Using an LC–QTOF platform, we performed qualitative and quantitative metabolomic analyses on 18 fecal samples, detecting a total of 10,509 peaks, of which 3720 metabolites were successfully annotated. To ensure data reliability, overall quality was rigorously assessed by evaluating the reproducibility of QC samples, the stability of intra‐group samples, and the discrimination between groups (Figures S2A–S2C).

**FIGURE 3:**
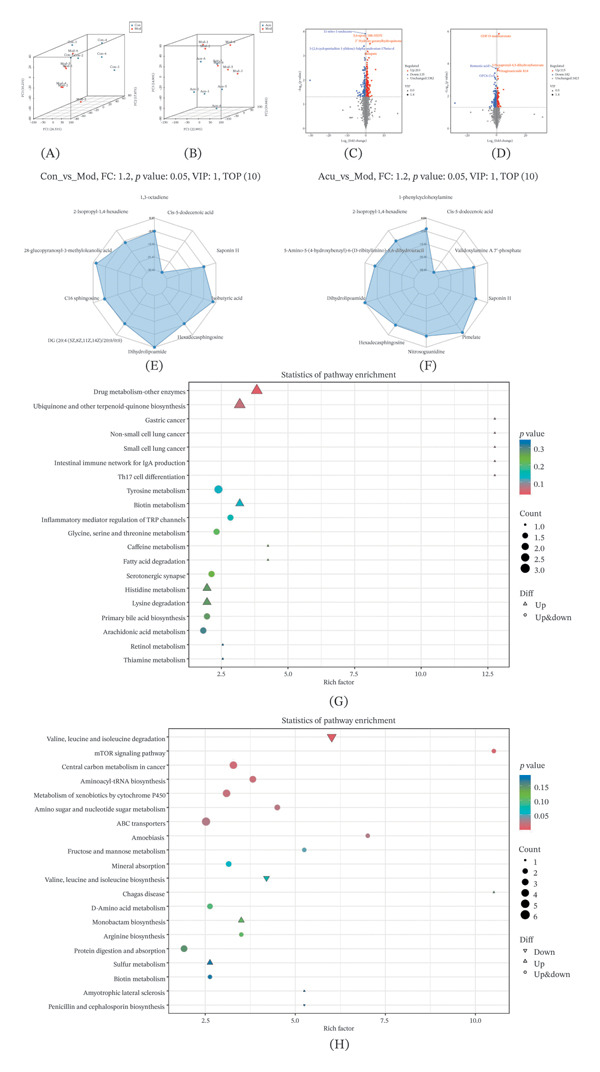
Metabolomics data analysis. (A) 3D PCA plot of Con vs. Mod groups; (B) 3D PCA plot of Mod vs. Acu groups; (C, D) volcano plots of differentially abundant metabolites between groups; (E, F) radar plots of representative top 10 differential metabolites; and (G, H) KEGG pathway enrichment analysis of differential metabolites between groups.

Principal component analysis (PCA) revealed clear separation among the Con, Mod, and Acu groups at the metabolomic level (Figure 3A,B), indicating that both disease modeling and acupuncture intervention induced measurable and distinct metabolic alterations. OPLS–DA further demonstrated robust group separation (Figures S2D–S2G). Between the Con and Mod groups, the model exhibited excellent goodness‐of‐fit (*R* ^2^ *Y* = 0.967) and strong predictive power (*Q* ^2^ *Y* = 0.565), confirming its ability to capture the metabolic distinctions with high accuracy and predictive validity. Similarly, the Mod vs. Acu comparison yielded a high *R* ^2^ *Y* value (0.961), with a slightly lower but still substantial predictive capacity, reflecting significant and predictable metabolic changes following acupuncture treatment.

Volcano plot screening, using the criteria of FC > 1.2 or < 0.83, *p* < 0.05, and VIP > 1, identified 338 differentially abundant metabolites (203 upregulated, 135 downregulated) between Con and Mod groups (Figure 3C; Table S5) and 297 metabolites (115 upregulated, 182 downregulated) between Mod and Acu groups (Figure 3D; Table S6). These results indicate that acupuncture may influence the metabolic profile in CM, particularly by affecting specific metabolic pathways.

Radar plots illustrated pronounced alterations among the top 10 differential metabolites across the groups. Specifically, comparison between the Con and Mod groups revealed significant changes in dihydrolipoamide, isobutyric acid, 28‐glucopyranosyl‐3‐methyloleanolic acid, C16 sphingosine, and saponin H (Figure 3E). In the comparison between the Mod and Acu groups, representative differential metabolites included dihydrolipoamide, pimelate, 1‐phenylcyclohexylamine, 5‐amino‐5‐(4‐hydroxybenzyl)‐6‐(D‐ribitylimino)‐5,6‐dihydrouracil, and nitrosoguanidine (Figure 3F). The observed shifts in their abundance imply that acupuncture may indirectly modulate central nervous system function by influencing these metabolic processes, thereby alleviating CM‐associated symptoms.

To further explore the biological significance of the differential metabolites, KEGG pathway enrichment analysis was performed. In the comparison between the Con and Mod groups, significantly enriched pathways included drug metabolism–other enzymes, ubiquinone and other terpenoid‐quinone biosynthesis, Th17 cell differentiation, and the intestinal immune network for IgA production (Figure 3G; Table S7). These results suggest that the CM model may induce disruptions in antioxidant capacity, as well as impair intestinal immune homeostasis and Th17 cell differentiation, potentially leading to altered neuroimmune regulation and neuroinflammation associated with chronic pain. In the comparison between the Mod and Acu groups, significantly enriched pathways included the degradation of valine, leucine, and isoleucine and the mTOR signaling pathway (Figure 3H; Table S8). Specifically, the modulation of branched‐chain amino acid (BCAA) degradation and aminoacyl‐tRNA biosynthesis suggests that acupuncture may restore neurometabolic homeostasis by regulating amino acid availability and protein synthesis. Enrichment of the mTOR signaling pathway and central carbon metabolism reflects recovery of cellular energy metabolism and neuronal function. Additionally. Taken together, these results indicate that acupuncture may exert its therapeutic effects in CM by selectively modulating specific metabolic pathways, thereby improving metabolic and neurofunctional disturbances at the molecular level.

### 3.4. Acupuncture Restores Gut Microbiota–Metabolite Interactions Implicated in CM Pathophysiology

LEfSe analysis revealed that the Acu group exhibited altered taxa predominantly clustered within specific lineages, suggesting that acupuncture induces directional and selective modulation of the gut microbiota in the CM model. Therefore, in the subsequent correlation analysis, we focused specifically on the relationships between metabolites and microbiota in the Acu and Mod groups. By integrating multidimensional data from differential metabolites and microbial communities, we aimed to comprehensively elucidate the complex interactions between gut microbiota and metabolites, thereby providing mechanistic insights into the potential therapeutic effects of acupuncture in CM.

**FIGURE 4:**
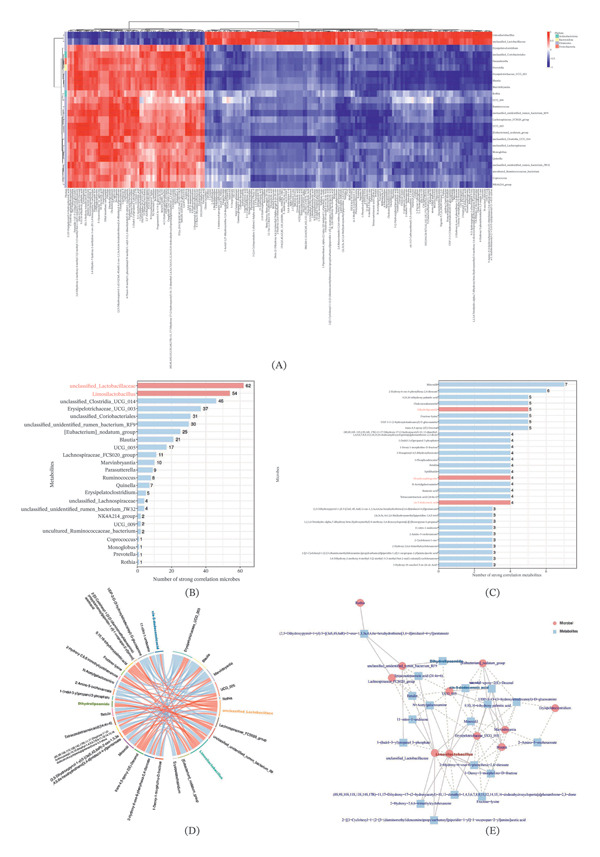
Correlation analysis between differential metabolites and differential microbiota. (A) Heatmap of metabolite–microbe correlations; (B, C) frequency bar plots of metabolite–microbe correlations; (D) chord diagram of metabolite–microbe correlations; and (E) network diagram of metabolite–microbe associations.

In the correlation heatmap (Figure 4A), *Limosilactobacillus* and unclassified_Lactobacillaceae displayed markedly distinct distribution patterns from other taxa, showing trends opposite to those of most microbial groups. This suggests that these two taxa may play unique roles within the gut microbial community. Moreover, both *Limosilactobacillus* and unclassified_Lactobacillaceae exhibited strong correlations (*p* < 0.01) with key differential metabolites between the Mod and Acu groups, such as cis‐5‐dodecenoic acid, dihydrolipoamide, pimelate, and hexadecasphingosine, highlighting their potential importance in metabolic regulation.

Further analysis using frequency bar plots of metabolite–microbe associations (Figure 4B,C) revealed that unclassified_Lactobacillaceae and *Limosilactobacillus* occurred at significantly higher frequencies than other taxa, underscoring their prominent roles in modulating CM‐related metabolites. Among the top 30 most frequently observed metabolites, dihydrolipoamide, hexadecasphingosine, and cis‐5‐dodecenoic acid were all enriched, with dihydrolipoamide ranking fifth, indicating its central position in the metabolic network.

The metabolite–microbe chord diagram (Figure 4D) clearly illustrated a significant association between unclassified_Lactobacillaceae and cis‐5‐dodecenoic acid, as well as a positive correlation between *Limosilactobacillus* and cis‐5‐dodecenoic acid and a negative correlation with dihydrolipoamide. This finding was further validated by the metabolite–microbe network analysis (Figure 4E), in which *Limosilactobacillus* emerged as a central network node, showing negative correlation with dihydrolipoamide and positive correlation with cis‐5‐dodecenoic acid.

### 3.5. *Lactobacillus* Supplementation Improves Pain‐Related Behaviors and Inflammatory Cytokine Expression in the Brain of CM Rats

Based on the preceding correlation analysis, we identified significant interactions between *Limosilactobacillus* and unclassified_Lactobacillaceae with key differential metabolites, with abundance changes strongly associated with the metabolomic profile of CM. This finding highlights the potential value of *Lactobacillus* taxa in CM intervention, particularly through the regulation of gut microbiota and metabolic pathways to synergistically alleviate chronic pain and neuroinflammation. Accordingly, we implemented exogenous supplementation with a mixed *Lactobacillus* preparation on top of the established CM model and acupuncture intervention to further validate its effects. Notably, *Limosilactobacillus* and *Lactobacillus murinus* belong to distinct clades within the Lactobacillaceae family, and our taxonomic analysis also revealed that CM‐associated dysbiosis involves significant changes across multiple species within the *Lactobacillus* genus, rather than being confined to a single species. Based on these characteristics, we selected a multistrain probiotic formulation composed of *Lactobacillus plantarum*, *Lactobacillus paracasei*, *Lactobacillus* acidophilus, and *Lactobacillus delbrueckii subsp. bulgaricus*. These strains are among the most widely used *Lactobacillus* representatives worldwide, with well‐established clinical safety and functional profiles. Previous studies have demonstrated that different *Lactobacillus* species share substantial functional similarities and exhibit complementary effects in maintaining gut barrier integrity, promoting SCFA production, modulating immune responses, and influencing gut–brain axis signaling [23–26]. Therefore, supplementation with multiple commonly used Lactobacillus species could not only mimic and amplify acupuncture’s modulatory effects on key *Lactobacillus* taxa at the functional level but also offer high reproducibility and strong potential for clinical translation.

**FIGURE 5:**
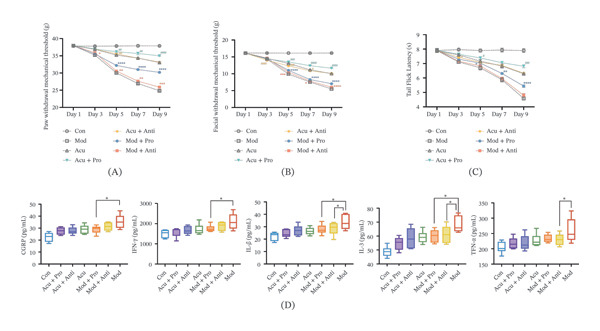
Effects of probiotic and antibiotic treatment on pain sensitivity and inflammatory cytokine levels in the brain of CM rats. (A) Comparison of PWMT under probiotic and antibiotic treatment. (B) Comparison of FWMT under probiotic and antibiotic treatment. (C) Comparison of TFL under probiotic and antibiotic treatment. (D) ELISA results for inflammatory cytokine levels in brain tissue under probiotic and antibiotic treatment (box plot representation). *n* = 6; compared with the mod: ^∗^ *p* < 0.05, ^∗∗^ *p* < 0.01, ^∗∗∗^ *p* < 0.001, ^∗∗∗∗^ *p* < 0.0001; compared with the Acu: ^#^ *p* < 0.05, ^##^ *p* < 0.01, ^###^ *p* < 0.001, ^####^ *p* < 0.0001.

We found that *Lactobacillus* supplementation significantly enhanced the analgesic effects of acupuncture. Compared with the Acu group, the Acu + Pro group exhibited greater improvements in PWMT and FWMT (*p* < 0.0001; Figure 5A,B, Tables S9 and S10), as well as significantly prolonged TFL (*p* < 0.001; Figure 5C, Table S11), suggesting that *Lactobacillus* may further potentiate the therapeutic effects of acupuncture in CM via modulation of the gut microbiota. Compared with the Mod group, *Lactobacillus* supplementation in the Mod + Pro group also significantly improved pain sensitivity (*p* < 0.0001), indicating that probiotic supplementation alone can effectively ameliorate pain‐related behaviors in the CM model. In the antibiotic‐treated groups, Mod + Anti rats, in which gut microbiota were depleted, showed only slight improvements in PWMT and FWMT (*p* < 0.05), with no significant improvement in TFL. Similarly, in the Acu + Anti group, antibiotic‐mediated depletion of the gut microbiota failed to produce significant pain relief and even resulted in intermittently lower pain thresholds than the Acu group during days 3–7 (*p* < 0.05), further underscoring the critical importance of gut microbial homeostasis.

ELISA results (Figure 5D) showed no significant differences in inflammatory cytokine expression between the Acu + Pro and Acu + Anti groups compared with the Acu group. This finding suggests that, under conditions where acupuncture has already markedly suppressed inflammatory responses, additional probiotic or antibiotic interventions do not further enhance its anti‐inflammatory effects. Interestingly, the Mod + Pro group exhibited significant differences from the Mod group in the expression levels of CGRP, IFN‐γ, IL‐1β, and IL‐3 (*p* < 0.05), indicating that probiotic supplementation alone can partially reverse model‐induced neuroinflammation and central sensitization. Although this effect may not fully match the magnitude of acupuncture or acupuncture combined with probiotics, the results further establish gut microbial homeostasis as a potential therapeutic target. In the Mod + Anti group, despite gut microbiota depletion by antibiotic treatment, IL‐1β, IL‐3, and TNF‐α levels remained significantly lower than those in the Mod group (*p* < 0.05), suggesting that gut microbial depletion per se can alter central inflammatory cytokine expression patterns. The underlying mechanism may involve the removal of pathogenic bacteria, the remodeling of gut microbial metabolite profiles, or the downregulation of immune response thresholds.

## 4. Discussion

CM is the second leading cause of disability‐adjusted life years globally, and studies have shown that patients often present with gut dysbiosis and metabolic disturbances [7, 8]. The MGBA tightly connects the gut microbiota with the central nervous system. The high plasticity of the gut microecology makes it an important therapeutic target for central nervous system disorders [27]. As an external treatment modality, acupuncture activates the neuroimmune network by stimulating specific acupoints, indirectly modulating gut permeability, microbiota structure, and metabolite levels, exhibiting neuroprotective and metabolic regulatory effects [20, 28, 29]. Therefore, by regulating the microbiota balance within the MGBA, acupuncture represents a potential therapeutic strategy for CM.

In this study, we systematically explored the effects of acupuncture on an NTG‐induced CM rat model. The experimental results first confirmed the stability and reliability of the model, demonstrating that NTG effectively induces sustained changes in pain perception within the central nervous system, consistent with previous reports [30]. The analgesic effects of acupuncture progressively enhanced with treatment duration, demonstrating its persistent efficacy in pain relief and its potential to improve central pain sensitization. ELISA results further indicated that NTG significantly activated central neuroinflammation, while acupuncture treatment significantly downregulated the expression of multiple inflammatory cytokines. Notably, although previous studies have reported the anti‐inflammatory properties of acupuncture, our findings specifically reveal its time‐dependent analgesic enhancement and targeted regulation of neuroinflammatory responses in a CM model. This suggests that acupuncture not only alleviates central inflammation but may also enhance analgesic effects by inhibiting neuroinflammation and immune responses, corroborating findings from previous studies [31, 32]. In conclusion, acupuncture alleviates central neuroinflammation and increases pain thresholds, effectively improving CM‐related central sensitization and providing new experimental evidence and theoretical support for its use in CM prevention and treatment.

In subsequent investigations, 16S rDNA sequencing showed that acupuncture significantly improved gut microbiota dysbiosis in CM rats. However, it is noteworthy that, as an exogenous intervention, acupuncture did not significantly alter the overall richness and evenness of the microbiota but likely affected gut–brain axis signaling and central nervous system responses by selectively modulating the abundance and functions of specific microbiota. Further phylogenetic and taxonomic analyses supported this hypothesis. Among key taxa, Akkermansia muciniphila and *Lactobacillus* species played central roles in the acupuncture effects. A. muciniphila, a crucial probiotic, maintains gut barrier integrity by degrading intestinal mucus layer polysaccharides, reducing the entry of proinflammatory factors into circulation, and thereby lowering systemic inflammation [33, 34]. Additionally, its metabolites, especially SCFAs, can reduce the secretion of proinflammatory cytokines via neuroimmune interactions [35, 36]. Acupuncture may therefore exert its antimigraine effects by increasing the abundance of A. muciniphila, enhancing gut barrier function, and reducing the transfer of gut‐derived toxins and pro‐inflammatory molecules to the central nervous system. Consistent with this, Wang et al. [37] observed abnormal changes in Akkermansiaceae in a CM animal model, while Xie et al. [38] further confirmed that acupuncture significantly increased the abundance of this taxon and inhibited the proinflammatory shift in gut microbiota. On the other hand, *Lactobacillus* species, as classic probiotics, regulate lipid metabolism and gut–brain axis function through SCFA production (such as butyrate), improve gut barrier integrity, and modulate local immune responses, indirectly alleviating neuroinflammation [39]. Numerous studies have emphasized the critical role of *Lactobacillus* in optimizing microbial structure, enhancing immune function, and protecting the nervous system [40, 41]. Notably, a reduction in *Lactobacillus* species is considered a significant marker of microbial changes in CM [42]; Mendelian randomization analysis also suggests a causal relationship between the genetic predisposition for higher *Lactobacillus* abundance and the occurrence of clinical migraine [43]. These findings collectively indicate that *Lactobacillus* plays a cross‐species role in the pathogenesis and regulation of CM, and its targeted modulation may become a key strategy for migraine intervention.

In the subsequent comprehensive analysis combining 16S rRNA gene sequencing and metabolomics, we aimed to further explore the mechanisms underlying acupuncture intervention in the CM model, with a particular focus on its role in regulating gut microbiota and metabolite profiles. Notably, correlation analysis revealed that *Limosilactobacillus* and unclassified_Lactobacillaceae may play unique and pivotal roles in the regulation of CM. Both belong to the Lactobacillaceae family and are important members of the *Lactobacillus* genus, providing a clear theoretical basis for our decision to administer exogenous mixed *Lactobacillus* supplementation. Further analysis revealed that these taxa exhibited significant correlations with several key metabolites, such as cis‐5‐dodecenoic acid and dihydrolipoamide, suggesting that *Lactobacillus* taxa are not only involved in lipid and energy metabolism remodeling but may also regulate central nervous system function through modulation of gut–brain axis signaling. Network analysis further confirmed that *Limosilactobacillus* is a central node in the microbiota–metabolite interaction network. This structural feature indicates that *Lactobacillus* taxa could be critical “hubs” linking metabolic pathways to pain phenotypes, potentially playing a decisive role in the improvement of CM symptoms.

Based on this hypothesis, we conducted exogenous mixed *Lactobacillus* supplementation experiments to verify its synergistic effects with acupuncture. The results showed that *Lactobacillus* supplementation significantly enhanced the analgesic effects of acupuncture. More importantly, the exogenous supplementation of *Lactobacillus* not only indirectly modulated the gut microbiota but also directly improved pain and inflammation symptoms [44, 45], even playing an active role in symptom relief in CM patients [46]. The inflammatory cytokine assays further supported this conclusion, suggesting that *Lactobacillus* enhances the effects of acupuncture by optimizing gut microbiota structure and function and may also exert analgesic effects through the direct inhibition of central nervous system inflammation. This mechanism aligns with previous research, which indicated that specific probiotics can significantly reduce neuroinflammation by modulating immune–inflammatory responses, thus alleviating chronic pain symptoms [47, 48]. In summary, *Lactobacillus* taxa have dual value in the compound treatment strategy for CM using acupuncture: on one hand, as key regulators of gut–brain axis metabolic pathways, they improve central nervous system function by targeting specific metabolite–microbe interaction networks; on the other hand, exogenous supplementation significantly amplifies the analgesic and anti‐inflammatory effects of acupuncture, providing strong theoretical and practical support for the comprehensive management of CM.

On the other hand, integrated functional prediction using PICRUSt2 and metabolomics analysis revealed a significant overlap in several key metabolic and signaling pathways, with particular emphasis on the amino acid metabolism network and the mTOR signaling pathway. In both the Con–Mod and Mod–Acu comparisons, key pathways including purine/pyrimidine metabolism, amino acid biosynthesis, carbon metabolism, and mTOR‐related pathways repeatedly appeared, suggesting that both the onset of CM and the effects of acupuncture intervention may depend on central regulation of material and energy metabolism. Among these pathways, changes in the mTOR signaling pathway are particularly noteworthy. mTOR, as a central hub integrating cellular energy status, nutrient signaling, and stress responses, exhibits bidirectional regulation with autophagy [49]. Previous studies have shown that CM patients exhibit elevated mTOR activity and decreased autophagy levels, which are closely associated with sustained neuroinflammation and pain sensitization [50]. In animal models, excessive mTOR activation inhibits autophagy, promotes abnormal protein aggregation, and triggers inflammasome activation, thereby exacerbating neuronal damage [51]. In this study, acupuncture significantly rebalanced the abnormal mTOR signaling axis in the CM model, suggesting that acupuncture may improve central microenvironment homeostasis by alleviating autophagy inhibition and promoting the clearance of metabolic waste and inflammatory mediators. This finding is consistent with previous research showing that acupuncture suppresses the mTOR pathway and enhances autophagy to alleviate neuroinflammation [52].

Another key intersection is amino acid metabolism and central energy homeostasis. BCAA degradation products provide carbon skeletons for the tricarboxylic acid (TCA) cycle, supplying energy to neurons [53]. Previous studies have found that BCAA metabolic abnormalities are closely associated with insufficient brain energy supply and the persistence of pain hypersensitivity [54]. Our results showed that acupuncture significantly restored the BCAA degradation pathway and related metabolite levels, suggesting that acupuncture may optimize the BCAA–TCA energy supply chain, indirectly meeting neuronal metabolic demands, and synergizing with mTOR–autophagy regulation. The multiomics analysis in this study revealed several key signaling and metabolic pathways associated with CM, providing multiple potential entry points for future research. Of course, the functions predicted by PICRUSt2 are only inferred. Although PICRUSt2 provides valuable predictive information, we should still interpret its results with caution. Subsequent work can be based on these findings to explore in greater depth the mechanisms of mTOR–autophagy regulation, amino acid–energy metabolism remodeling, and their roles within the gut–brain axis.

The limitations of our study lie in the fact that we only preliminarily explored and validated the key role of *Lactobacillus* in the pathological changes of CM and acupuncture treatment, as well as the positive impact of exogenous *Lactobacillus* supplementation on the disease. However, we have not yet deeply investigated its mechanisms in microbiota interaction networks, metabolite signaling, and immune regulation. Future studies could integrate techniques such as metatranscriptomics, single‐cell sequencing, and metabolic flux analysis to systematically investigate the causal relationship between *Lactobacillus* and host immune–metabolic interactions, providing more rigorous preclinical data to elucidate the connections between acupuncture, gut microecology, and central nervous function.

## 5. Conclusion

This study combined behavioral testing, molecular assays, 16S rDNA sequencing, and metabolomics to investigate the effects and mechanisms of acupuncture in CM rats. Acupuncture significantly alleviated pain hypersensitivity and central inflammation and restored gut microbiota balance, notably enriching Lactobacillaceae. Metabolomic and microbiota–metabolite network analyses identified cis‐5‐dodecenoic acid and dihydrolipoamide as key metabolites closely associated with Lactobacillaceae, suggesting that acupuncture may modulate gut–brain axis signaling through lactobacilli‐related metabolic pathways. Supplementation with mixed *Lactobacillus* further confirmed the importance of gut homeostasis in CM intervention and its capacity to improve pain and inflammation.

Overall, acupuncture offers multiple benefits in CM management, including suppression of neuroinflammation, reversal of gut dysbiosis, and alleviation of pain sensitization, potentially via the *Lactobacillus*–metabolite–inflammation axis, providing a mechanistic basis for clinical translation.

**Nomenclature**
CM: Chronic migraine
LPS: Lipopolysaccharide
SCFA: Short‐chain fatty acid
HPA: Hypothalamic–pituitary–adrenal
NTG: Nitroglycerin
PWMT: Paw withdrawal mechanical threshold
FWMT: Facial withdrawal mechanical threshold
TFL: Tail‐flick latency
Sp5c: Spinal trigeminal nucleus caudalis
CGRP: Calcitonin gene‐related peptide
IFN‐γ: Interferon‐gamma
IL‐1β: Interleukin‐1 beta
IL‐3: Interleukin‐3
TNF‐α: Tumor necrosis factor‐alpha
LEfSe: Linear discriminant analysis effect size
VIP: Variable importance in projection
FC: Fold change
PCoA: Principal coordinates analysis
NMDS: Nonmetric multidimensional scaling
QC: Quality control
PCA: Principal component analysis
MGBA: Microbiota–gut–brain axis
BCAA: Branched‐chain amino acid
TCA: Tricarboxylic acid
KEGG: Kyoto Encyclopedia of Genes and Genomes

## Author Contributions

All authors contributed significantly to this study. Shiqi Sun and Longyao Xu jointly conceived and designed the overall research framework. Yuyan Wang and Shuangyuan Hu were responsible for conducting the animal experiments and collecting biological samples. Shiqi Sun performed the metabolomics analysis as well as gut microbiota sequencing and bioinformatics processing. Longyao Xu and Yuyan Wang carried out data curation and statistical analysis, with Shuangyuan Hu assisting in data visualization and figure preparation. The initial draft of the manuscript was written by Shiqi Sun, and all authors—particularly Mingsheng Sun, Jing Yuan, and Ling Zhao—participated in revising, reviewing, and polishing the manuscript. Ling Zhao provided overall guidance for the study and was responsible for securing project funding.

## Funding

This study was supported by the China National Natural Science Foundation (Nos. 82274664 and 82430124).

## Disclosure

All authors have read and approved the final version of the manuscript.

## Ethics Statement

All experimental procedures were approved by the Experimental Animal Ethics Committee of Chengdu University of Traditional Chinese Medicine (Approval No. 2019‐23) and were conducted in accordance with the Guidelines for the Care and Use of Laboratory Animals of the Chinese Pharmacological Society.

## Consent

The authors have nothing to report.

## Conflicts of Interest

The authors declare no conflicts of interest.

## Supporting Information

Additional supporting information can be found online in the Supporting Information section.

## Supporting information


**Supporting Information 1** Supporting Figure S1: α‐diversity analysis of the microbiota. This figure presents four commonly used α‐diversity indices (Chao1, Shannon, Simpson, and ACE; panels A–D), calculated for each sample to assess within‐sample richness and diversity across experimental groups, together with the corresponding statistical comparisons.

**Supporting Information 2** Supporting Figure S2: Additional results for data reliability and multivariate model assessment. Panels A–C evaluate the reliability of the metabolomics dataset by examining the reproducibility of quality control (QC) samples, the stability/consistency of intra‐group samples, and the discrimination between groups. Panels D–G show OPLS–DA results illustrating disease model–associated metabolic alterations and group separation induced by acupuncture intervention, along with the model performance/validation outputs reported in the figure.

**Supporting Information 3** Supporting Table S1: Statistical analysis of mechanical paw withdrawal thresholds (three‐group comparison). This table provides group‐wise results for mechanical paw withdrawal thresholds, including descriptive statistics and between‐group comparisons, corresponding to the behavioral assessments described in the main text.

**Supporting Information 4** Supporting Table S2: Statistical analysis of mechanical facial withdrawal thresholds (three‐group comparison). This table reports the statistical analysis of mechanical facial withdrawal thresholds, including descriptive statistics and between‐group comparisons.

**Supporting Information 5** Supporting Table S3: Statistical analysis of tail‐flick latency (three‐group comparison). This table reports the statistical analysis of tail‐flick latency, including descriptive statistics and between‐group comparisons.

**Supporting Information 6** Supporting Table S4: Class‐level taxa analysis. This table summarizes one‐way ANOVA results for taxa at the class level, including relative abundance metrics and the associated statistical outputs for group comparisons.

**Supporting Information 7** Supporting Table S5: Differential metabolites (Con vs Mod). This table lists the 338 differential metabolites identified between the control (Con) and model (Mod) groups, together with the relevant statistical parameters reported by the differential analysis workflow.

**Supporting Information 8** Supporting Table S6: Differential metabolites (Mod vs Acu). This table lists the 297 differential metabolites identified between the model (Mod) and acupuncture (Acu) groups, together with the relevant statistical parameters reported by the differential analysis workflow.

**Supporting Information 9** Supporting Table S7: Pathway enrichment analysis (Con vs Mod). This table reports significantly enriched metabolic pathways between the Con and Mod groups, including enrichment statistics and pathway‐level summary metrics.

**Supporting Information 10** Supporting Table S8: Pathway enrichment analysis (Mod vs Acu). This table reports significantly enriched metabolic pathways between the Mod and Acu groups, including enrichment statistics and pathway‐level summary metrics.

**Supporting Information 11** Supporting Table S9: Statistical analysis of mechanical paw withdrawal thresholds (seven‐group comparison). This table provides the statistical analysis of mechanical paw withdrawal thresholds for the expanded seven‐group comparison, including descriptive statistics and between‐group comparisons.

**Supporting Information 12** Supporting Table S10: Statistical analysis of mechanical facial withdrawal thresholds (seven‐group comparison). This table provides the statistical analysis of mechanical facial withdrawal thresholds for the expanded seven‐group comparison, including descriptive statistics and between‐group comparisons.

**Supporting Information 13** Supporting Table S11: Statistical analysis of tail‐flick latency (seven‐group comparison). This table provides the statistical analysis of tail‐flick latency for the expanded seven‐group comparison, including descriptive statistics and between‐group comparisons.

## Data Availability

Data are available on request due to privacy/ethical restrictions.
