# Supplementary figures and images for: Acupuncture Alleviates Neuroinflammation in Chronic Migraine by Modulating Lactobacillus and Its Metabolite Pathways
Source: Pain Res Manag. 2026 Jun 23;2026:5189419. doi: 10.1155/prm/5189419 (PMC13287961; doi:10.1155/prm/5189419)

**A**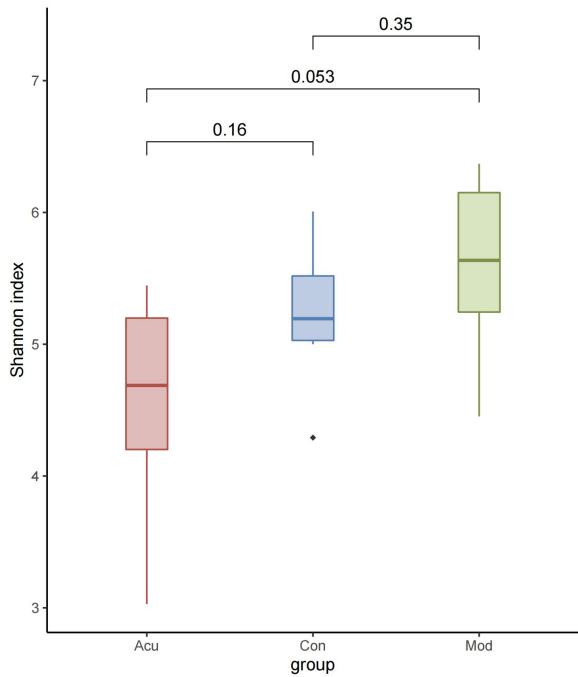**B**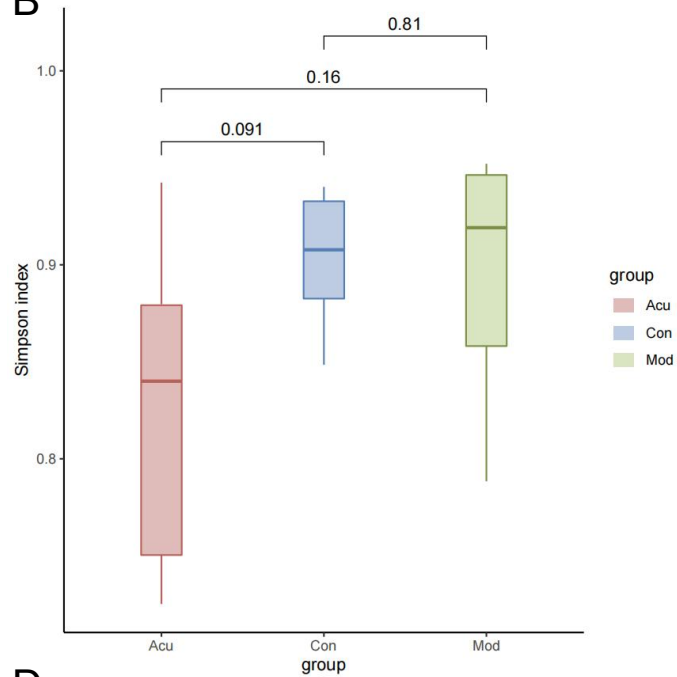**C**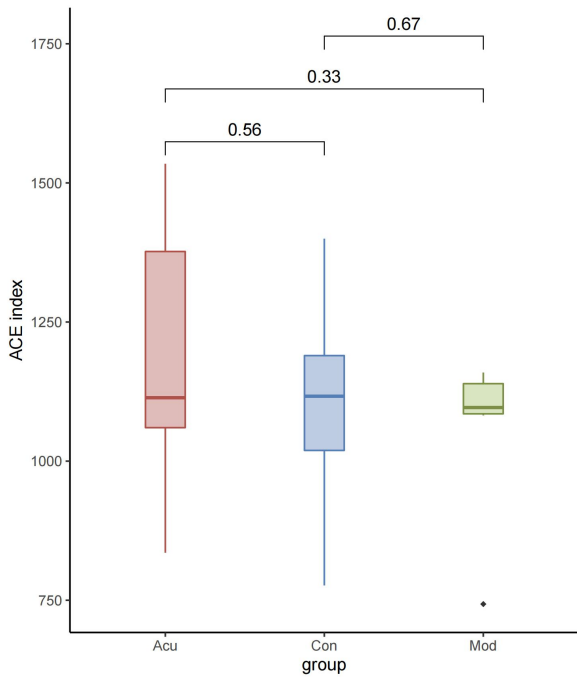**D**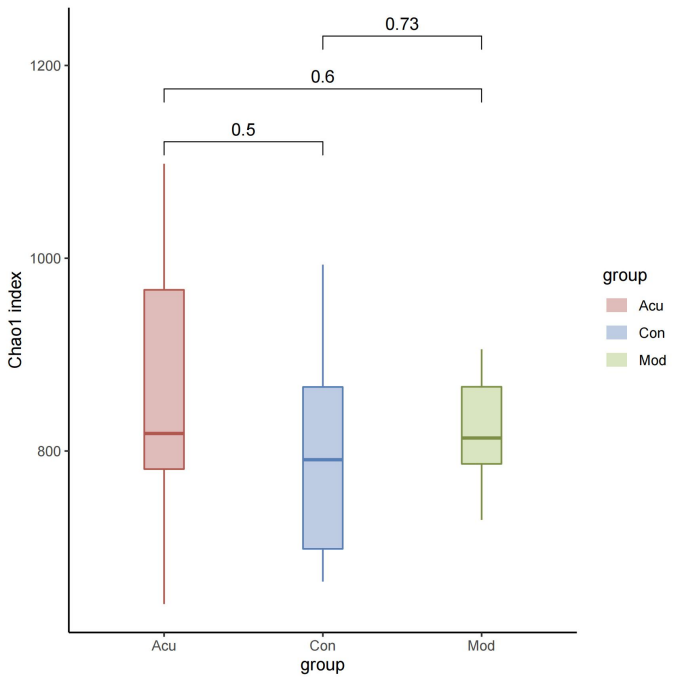

Supplement: Supplementary file 1 — Supporting Information 1 Supporting Figure S1: α‐diversity analysis of the microbiota. This figure presents four commonly used α‐diversity indices (Chao1, Shannon, Simpson, and ACE; panels A–D), calculated for each sample to assess within‐sample richness and diversity across experimental groups, together with the corresponding statistical comparisons. [file PRM-2026-5189419-s012.pdf]

A

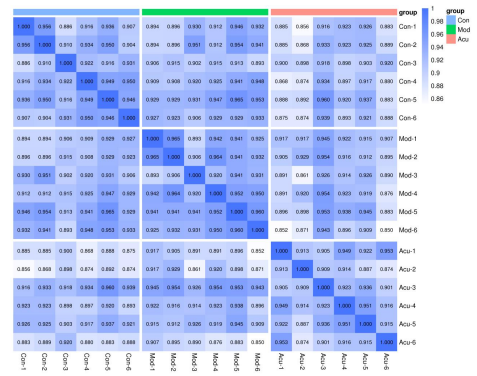

B

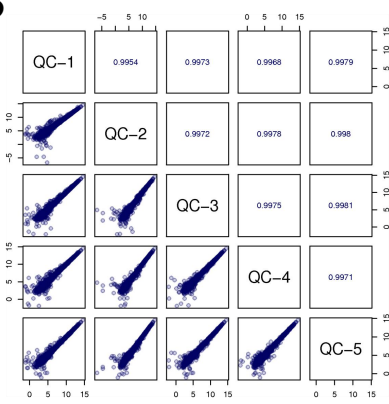

C

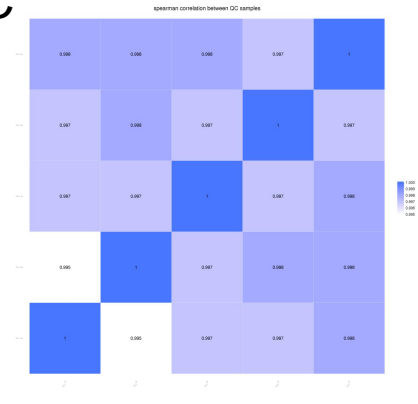

D

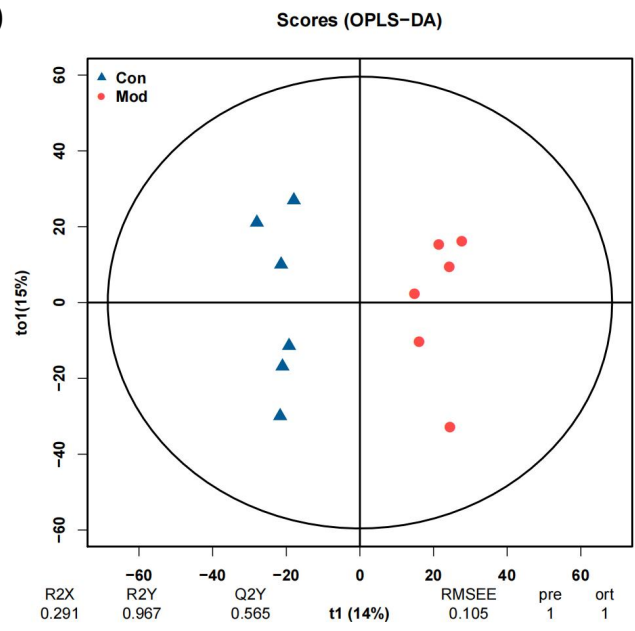

E

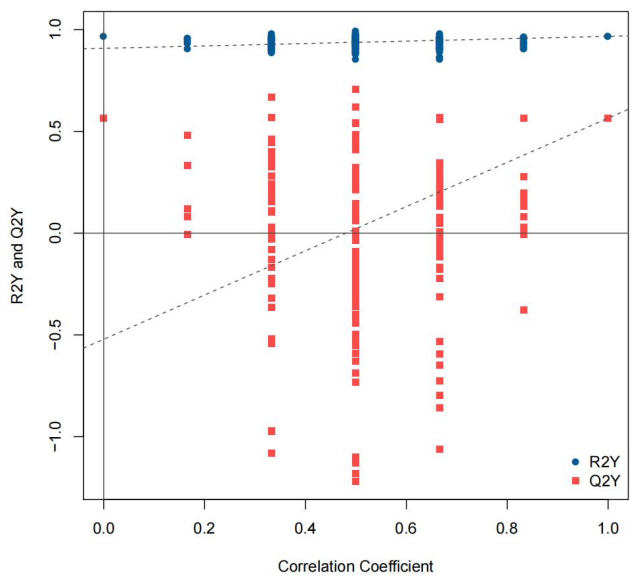

E

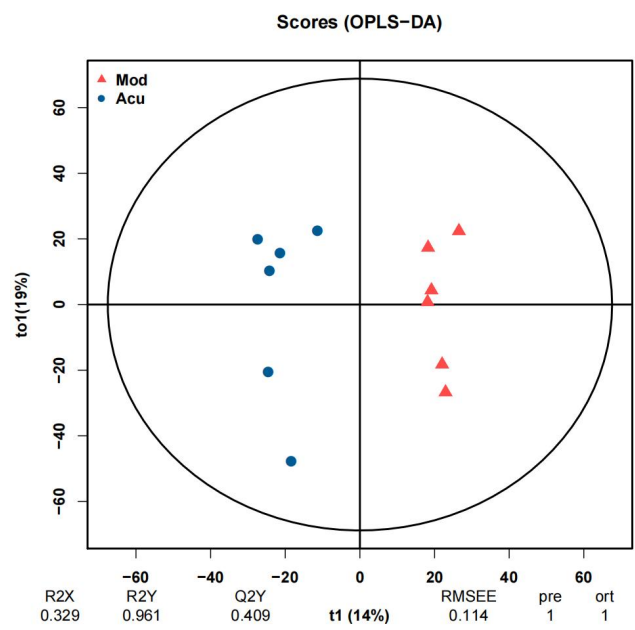

F

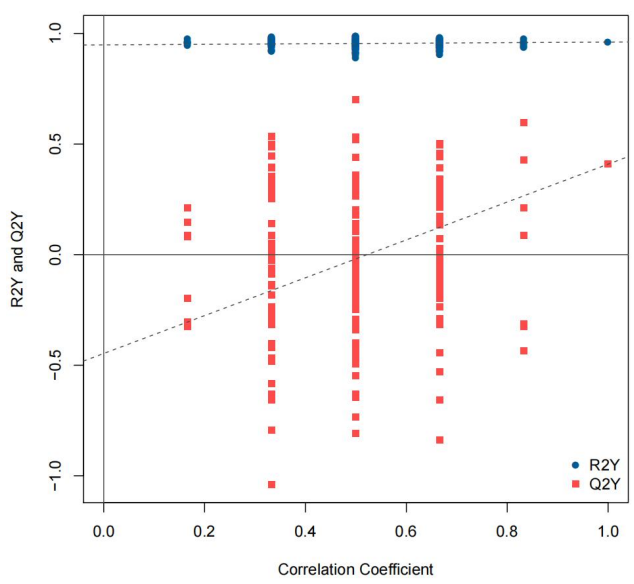

Supplement: Supplementary file 2 — Supporting Information 2 Supporting Figure S2: Additional results for data reliability and multivariate model assessment. Panels A–C evaluate the reliability of the metabolomics dataset by examining the reproducibility of quality control (QC) samples, the stability/consistency of intra‐group samples, and the discrimination between groups. Panels D–G show OPLS–DA results illustrating disease model–associated metabolic alterations and group separation induced by acupuncture intervention, along with the model performance/validation outputs reported in the figure. [file PRM-2026-5189419-s001.pdf]
