# Supplementary material for: Acupuncture Alleviates Neuroinflammation in Chronic Migraine by Modulating Lactobacillus and Its Metabolite Pathways
Source: Pain Res Manag. 2026 Jun 23;2026:5189419. doi: 10.1155/prm/5189419 (PMC13287961; doi:10.1155/prm/5189419)
Supplement: Supplementary file 3 — Supporting Information 3 Supporting Table S1: Statistical analysis of mechanical paw withdrawal thresholds (three‐group comparison). This table provides group‐wise results for mechanical paw withdrawal thresholds, including descriptive statistics and between‐group comparisons, corresponding to the behavioral assessments described in the main text. [file PRM-2026-5189419-s002.docx]

**Table S1** Statistical analysis of mechanical paw withdrawal thresholds.

| **Tukey's multiple comparisons test** | **Mean diff.** | **95.00% CI of diff.** | **Below threshold?** | **Summary** | **Adjusted *P* Value** |
| --- | --- | --- | --- | --- | --- |
| Day1 | | | | | |
| Con vs. Mod | -0.1067 | -0.5440 to 0.3307 | No | ns | 0.7848 |
| Con vs. Acu | -0.1083 | -0.5020 to 0.2853 | No | ns | 0.7106 |
| Mod vs. Acu | -0.001667 | -0.3383 to 0.3350 | No | ns | 0.9999 |
|  |  |  |  |  |  |
| Day3 | | | | | |
| Con vs. Mod | 2.577 | 2.215 to 2.938 | Yes | **** | <0.0001 |
| Con vs. Acu | 0.8833 | 0.5580 to 1.209 | Yes | **** | <0.0001 |
| Mod vs. Acu | -1.693 | -2.078 to -1.308 | Yes | **** | <0.0001 |
|  |  |  |  |  |  |
| Day5 | | | | | |
| Con vs. Mod | 7.803 | 7.475 to 8.132 | Yes | **** | <0.0001 |
| Con vs. Acu | 2.603 | 2.282 to 2.925 | Yes | **** | <0.0001 |
| Mod vs. Acu | -5.2 | -5.422 to -4.978 | Yes | **** | <0.0001 |
|  |  |  |  |  |  |
| Day7 | | | | | |
| Con vs. Mod | 10.94 | 10.51 to 11.37 | Yes | **** | <0.0001 |
| Con vs. Acu | 3.547 | 3.146 to 3.948 | Yes | **** | <0.0001 |
| Mod vs. Acu | -7.397 | -7.775 to -7.019 | Yes | **** | <0.0001 |
|  |  |  |  |  |  |
| Day9 | | | | | |
| Con vs. Mod | 13.02 | 12.68 to 13.36 | Yes | **** | <0.0001 |
| Con vs. Acu | 4.82 | 4.479 to 5.161 | Yes | **** | <0.0001 |
| Mod vs. Acu | -8.2 | -8.562 to -7.838 | Yes | **** | <0.0001 |
