# Supplementary material for: Acupuncture Alleviates Neuroinflammation in Chronic Migraine by Modulating Lactobacillus and Its Metabolite Pathways
Source: Pain Res Manag. 2026 Jun 23;2026:5189419. doi: 10.1155/prm/5189419 (PMC13287961; doi:10.1155/prm/5189419)
Supplement: Supplementary file 4 — Supporting Information 4 Supporting Table S2: Statistical analysis of mechanical facial withdrawal thresholds (three‐group comparison). This table reports the statistical analysis of mechanical facial withdrawal thresholds, including descriptive statistics and between‐group comparisons. [file PRM-2026-5189419-s003.docx]

**Table S2** Statistical analysis of mechanical facial withdrawal thresholds.

| **Tukey's multiple comparisons test** | **Mean diff.** | **95.00% CI of diff.** | **Below threshold?** | **Summary** | **Adjusted *P* Value** |
| --- | --- | --- | --- | --- | --- |
| Day1 | | | | | |
| Con vs. Mod | 0.02833 | -0.05494 to 0.1116 | No | ns | 0.6152 |
| Con vs. Acu | 0.025 | -0.07456 to 0.1246 | No | ns | 0.7753 |
| Mod vs. Acu | -0.003333 | -0.08969 to 0.08302 | No | ns | 0.9933 |
|  |  |  |  |  |  |
| Day3 | | | | | |
| Con vs. Mod | 1.78 | 1.599 to 1.961 | Yes | **** | <0.0001 |
| Con vs. Acu | 1.552 | 1.381 to 1.723 | Yes | **** | <0.0001 |
| Mod vs. Acu | -0.2283 | -0.3488 to -0.1079 | Yes | ** | 0.0018 |
|  |  |  |  |  |  |
| Day5 | | | | | |
| Con vs. Mod | 6.217 | 6.096 to 6.337 | Yes | **** | <0.0001 |
| Con vs. Acu | 3.062 | 2.929 to 3.194 | Yes | **** | <0.0001 |
| Mod vs. Acu | -3.155 | -3.306 to -3.004 | Yes | **** | <0.0001 |
|  |  |  |  |  |  |
| Day7 | | | | | |
| Con vs. Mod | 8.577 | 8.402 to 8.751 | Yes | **** | <0.0001 |
| Con vs. Acu | 4.942 | 4.820 to 5.064 | Yes | **** | <0.0001 |
| Mod vs. Acu | -3.635 | -3.805 to -3.465 | Yes | **** | <0.0001 |
|  |  |  |  |  |  |
| Day9 | | | | | |
| Con vs. Mod | 10.64 | 10.47 to 10.81 | Yes | **** | <0.0001 |
| Con vs. Acu | 6.11 | 5.940 to 6.280 | Yes | **** | <0.0001 |
| Mod vs. Acu | -4.533 | -4.616 to -4.451 | Yes | **** | <0.0001 |
