# Supplementary material for: Acupuncture Alleviates Neuroinflammation in Chronic Migraine by Modulating Lactobacillus and Its Metabolite Pathways
Source: Pain Res Manag. 2026 Jun 23;2026:5189419. doi: 10.1155/prm/5189419 (PMC13287961; doi:10.1155/prm/5189419)
Supplement: Supplementary file 5 — Supporting Information 5 Supporting Table S3: Statistical analysis of tail‐flick latency (three‐group comparison). This table reports the statistical analysis of tail‐flick latency, including descriptive statistics and between‐group comparisons. [file PRM-2026-5189419-s004.docx]

**Table S3** Statistical analysis of tail-flick latency.

| **Tukey's multiple comparisons test** | **Mean diff.** | **95.00% CI of diff.** | **Below threshold?** | **Summary** | **Adjusted *P* Value** |
| --- | --- | --- | --- | --- | --- |
| Day1 | | | | | |
| Con vs. Mod | 0.006667 | -0.2155 to 0.2289 | No | ns | 0.9963 |
| Con vs. Acu | 0.01667 | -0.1992 to 0.2325 | No | ns | 0.9756 |
| Mod vs. Acu | 0.01 | -0.2026 to 0.2226 | No | ns | 0.9909 |
|  |  |  |  |  |  |
| Day3 | | | | | |
| Con vs. Mod | 0.8567 | 0.7321 to 0.9812 | Yes | **** | <0.0001 |
| Con vs. Acu | 0.365 | 0.2585 to 0.4715 | Yes | **** | <0.0001 |
| Mod vs. Acu | -0.4917 | -0.6221 to -0.3613 | Yes | **** | <0.0001 |
|  |  |  |  |  |  |
| Day5 | | | | | |
| Con vs. Mod | 1.193 | 0.9807 to 1.406 | Yes | **** | <0.0001 |
| Con vs. Acu | 0.7367 | 0.5586 to 0.9148 | Yes | **** | <0.0001 |
| Mod vs. Acu | -0.4567 | -0.6720 to -0.2414 | Yes | *** | 0.0005 |
|  |  |  |  |  |  |
| Day7 | | | | | |
| Con vs. Mod | 2.057 | 1.833 to 2.280 | Yes | **** | <0.0001 |
| Con vs. Acu | 1.103 | 0.8833 to 1.323 | Yes | **** | <0.0001 |
| Mod vs. Acu | -0.9533 | -1.190 to -0.7165 | Yes | **** | <0.0001 |
|  |  |  |  |  |  |
| Day9 | | | | | |
| Con vs. Mod | 3.312 | 3.131 to 3.492 | Yes | **** | <0.0001 |
| Con vs. Acu | 1.602 | 1.434 to 1.769 | Yes | **** | <0.0001 |
| Mod vs. Acu | -1.71 | -1.836 to -1.584 | Yes | **** | <0.0001 |
