# Supplementary material for: Acupuncture Alleviates Neuroinflammation in Chronic Migraine by Modulating Lactobacillus and Its Metabolite Pathways
Source: Pain Res Manag. 2026 Jun 23;2026:5189419. doi: 10.1155/prm/5189419 (PMC13287961; doi:10.1155/prm/5189419)
Supplement: Supplementary file 6 — Supporting Information 6 Supporting Table S4: Class‐level taxa analysis. This table summarizes one‐way ANOVA results for taxa at the class level, including relative abundance metrics and the associated statistical outputs for group comparisons. [file PRM-2026-5189419-s013.docx]

| **class** | **Acu**  **(Mean)** | **Acu**  **(Sd)** | **Con**  **(Mean)** | **Con**  **(Sd)** | **Mod**  (Mean) | **Mod**  (Sd) | **multiGroup**  (p) | **multiGroup**  (p-corrected) | **A:C**  (p) | **A:M**  (p) | **C:M** (p) |
| --- | --- | --- | --- | --- | --- | --- | --- | --- | --- | --- | --- |
| Actinobacteria | 0.005377 | 0.011884 | 0.000645 | 0.000747 | 0.001568 | 0.00134 | 0.472418 | 0.690458 | - | - | - |
| Alphaproteobacteria | 0.000155 | 0.00038 | 0.000204 | 0.000367 | 0.000027 | 0.000065 | 0.596829 | 0.755984 | - | - | - |
| Bacilli | 0.544996 | 0.089001 | 0.472865 | 0.056175 | 0.378462 | 0.07263 | 0.005057 | 0.048044 | >= 0.1 | < 0.01 | >= 0.1 |
| Bacteroidia | 0.223018 | 0.07224 | 0.319584 | 0.059324 | 0.289907 | 0.108367 | 0.150983 | 0.478111 | - | - | - |
| Campylobacteria | 0.000834 | 0.001146 | 0.002148 | 0.001519 | 0.000466 | 0.000676 | 0.058532 | 0.278026 | - | - | - |
| Clostridia | 0.110521 | 0.045964 | 0.138164 | 0.039047 | 0.300921 | 0.105037 | 0.000533 | 0.010131 | >= 0.1 | < 0.001 | < 0.01 |
| Coriobacteriia | 0.000447 | 0.000383 | 0.00031 | 0.000274 | 0.00124 | 0.000656 | 0.007238 | 0.045843 | >= 0.1 | < 0.05 | < 0.01 |
| Deferribacteres | 0.000077 | 0.000128 | 0.000119 | 0.000291 | 0.000025 | 0.000062 | 0.693776 | 0.775397 | - | - | - |
| Desulfovibrionia | 0.002877 | 0.00258 | 0.002638 | 0.001953 | 0.001767 | 0.003829 | 0.786282 | 0.829965 | - | - | - |
| Elusimicrobia | 0 | 0 | 0.000048 | 0.000074 | 0.000073 | 0.00008 | 0.158359 | 0.429831 | - | - | - |
| Gammaproteobacteria | 0.020944 | 0.022127 | 0.012501 | 0.011522 | 0.0151 | 0.009232 | 0.630511 | 0.748732 | - | - | - |
| Gemmatimonadetes | 0 | 0 | 0.000048 | 0.000074 | 0 | 0 | 0.115607 | 0.439305 | - | - | - |
| Kiritimatiellae | 0 | 0 | 0.000078 | 0.000192 | 0 | 0 | 0.391127 | 0.825712 | - | - | - |
| Methanobacteria | 0.000026 | 0.000063 | 0 | 0 | 0 | 0 | 0.391127 | 0.743141 | - | - | - |
| Negativicutes | 0.004866 | 0.005628 | 0.007978 | 0.008857 | 0.008146 | 0.01397 | 0.819977 | 0.819977 | - | - | - |
| Saccharimonadia | 0.001331 | 0.001028 | 0.000483 | 0.000347 | 0.001104 | 0.001282 | 0.320116 | 0.760275 | - | - | - |
| Spirochaetia | 0 | 0 | 0 | 0 | 0.000026 | 0.000063 | 0.391127 | 0.675583 | - | - | - |
| Vampirivibrionia | 0.000574 | 0.000339 | 0.000555 | 0.000475 | 0.000305 | 0.000381 | 0.453813 | 0.718537 | - | - | - |
| Verrucomicrobiae | 0.083959 | 0.180994 | 0.041634 | 0.088705 | 0.000863 | 0.001402 | 0.48271 | 0.655107 | - | - | - |

**Table S4** One-way ANOVA analysis of taxa at the class level.
