# Supplementary material for: Acupuncture Alleviates Neuroinflammation in Chronic Migraine by Modulating Lactobacillus and Its Metabolite Pathways
Source: Pain Res Manag. 2026 Jun 23;2026:5189419. doi: 10.1155/prm/5189419 (PMC13287961; doi:10.1155/prm/5189419)
Supplement: Supplementary file 11 — Supporting Information 11 Supporting Table S9: Statistical analysis of mechanical paw withdrawal thresholds (seven‐group comparison). This table provides the statistical analysis of mechanical paw withdrawal thresholds for the expanded seven‐group comparison, including descriptive statistics and between‐group comparisons. [file PRM-2026-5189419-s009.docx]

**Table S9** Statistical analysis of mechanical paw withdrawal thresholds（7 groups）.

| **Tukey's multiple comparisons test** | **Mean diff.** | **95.00% CI of diff.** | **Below threshold?** | **Summary** | **Adjusted *P* Value** |
| --- | --- | --- | --- | --- | --- |
| Day1 | | | | | |
| Con vs. Mod | -0.1067 | -0.6854 to 0.4721 | No | ns | 0.9914 |
| Mod vs. Acu | -0.001667 | -0.4519 to 0.4486 | No | ns | >0.9999 |
| Mod vs. Mod+Pro | -0.045 | -0.5406 to 0.4506 | No | ns | 0.9998 |
| Mod vs. Mod+Anti | -0.055 | -0.5010 to 0.3910 | No | ns | 0.9981 |
| Acu vs. Acu+Pro | -0.07167 | -0.3732 to 0.2299 | No | ns | 0.9709 |
| Acu vs. Acu+Anti | 0.01833 | -0.4017 to 0.4383 | No | ns | >0.9999 |
| Day3 | | | | | |
| Con vs. Mod | 2.577 | 2.097 to 3.057 | Yes | **** | <0.0001 |
| Mod vs. Acu | -1.693 | -2.203 to -1.184 | Yes | **** | <0.0001 |
| Mod vs. Mod+Pro | -0.3583 | -0.8259 to 0.1092 | No | ns | 0.1602 |
| Mod vs. Mod+Anti | -0.7083 | -1.259 to -0.1579 | Yes | * | 0.0108 |
| Acu vs. Acu+Pro | -0.07667 | -0.6383 to 0.4850 | No | ns | 0.9982 |
| Acu vs. Acu+Anti | -0.02167 | -0.4638 to 0.4204 | No | ns | >0.9999 |
| Day5 | | | | | |
| Con vs. Mod | 7.803 | 7.365 to 8.241 | Yes | **** | <0.0001 |
| Mod vs. Acu | -5.2 | -5.494 to -4.906 | Yes | **** | <0.0001 |
| Mod vs. Mod+Pro | -2.187 | -2.483 to -1.890 | Yes | **** | <0.0001 |
| Mod vs. Mod+Anti | -0.54 | -0.8993 to -0.1807 | Yes | ** | 0.0038 |
| Acu vs. Acu+Pro | -0.9767 | -1.535 to -0.4180 | Yes | ** | 0.0028 |
| Acu vs. Acu+Anti | -0.365 | -0.6402 to -0.08982 | Yes | ** | 0.0088 |
| Day7 | | | | | |
| Con vs. Mod | 10.94 | 10.38 to 11.51 | Yes | **** | <0.0001 |
| Mod vs. Acu | -7.397 | -7.897 to -6.896 | Yes | **** | <0.0001 |
| Mod vs. Mod+Pro | -4.053 | -4.535 to -3.571 | Yes | **** | <0.0001 |
| Mod vs. Mod+Anti | -0.675 | -1.136 to -0.2141 | Yes | ** | 0.0072 |
| Acu vs. Acu+Pro | -1.337 | -2.060 to -0.6130 | Yes | ** | 0.0014 |
| Acu vs. Acu+Anti | 0.03333 | -0.3487 to 0.4154 | No | ns | 0.9996 |
| Day9 | | | | | |
| Con vs. Mod | 13.02 | 12.58 to 13.46 | Yes | **** | <0.0001 |
| Mod vs. Acu | -8.2 | -8.678 to -7.722 | Yes | **** | <0.0001 |
| Mod vs. Mod+Pro | -5.337 | -5.761 to -4.912 | Yes | **** | <0.0001 |
| Mod vs. Mod+Anti | -1.048 | -1.452 to -0.6447 | Yes | *** | 0.0001 |
| Acu vs. Acu+Pro | -1.963 | -2.421 to -1.506 | Yes | **** | <0.0001 |
| Acu vs. Acu+Anti | -0.1067 | -0.5181 to 0.3048 | No | ns | 0.9259 |
