# Supplementary material for: Acupuncture Alleviates Neuroinflammation in Chronic Migraine by Modulating Lactobacillus and Its Metabolite Pathways
Source: Pain Res Manag. 2026 Jun 23;2026:5189419. doi: 10.1155/prm/5189419 (PMC13287961; doi:10.1155/prm/5189419)
Supplement: Supplementary file 12 — Supporting Information 12 Supporting Table S10: Statistical analysis of mechanical facial withdrawal thresholds (seven‐group comparison). This table provides the statistical analysis of mechanical facial withdrawal thresholds for the expanded seven‐group comparison, including descriptive statistics and between‐group comparisons. [file PRM-2026-5189419-s010.docx]

**Table S10** Statistical analysis of mechanical facial withdrawal thresholds（7 groups）.

| **Tukey's multiple comparisons test** | **Mean diff.** | **95.00% CI of diff.** | **Below threshold?** | **Summary** | **Adjusted *P* Value** |
| --- | --- | --- | --- | --- | --- |
| Day1 | | | | | |
| Con vs. Mod | 0.02833 | -0.08282 to 0.1395 | No | ns | 0.9483 |
| Mod vs. Acu | -0.003333 | -0.1187 to 0.1121 | No | ns | >0.9999 |
| Mod vs. Mod+Pro | -0.008333 | -0.1510 to 0.1343 | No | ns | >0.9999 |
| Mod vs. Mod+Anti | -0.008333 | -0.09793 to 0.08126 | No | ns | 0.9998 |
| Acu vs. Acu+Pro | -0.015 | -0.1479 to 0.1179 | No | ns | 0.9994 |
| Acu vs. Acu+Anti | -0.015 | -0.1367 to 0.1067 | No | ns | 0.999 |
| Day3 | | | | | |
| Con vs. Mod | 1.78 | 1.540 to 2.020 | Yes | **** | <0.0001 |
| Mod vs. Acu | -0.2283 | -0.3897 to -0.06696 | Yes | ** | 0.0076 |
| Mod vs. Mod+Pro | -0.1317 | -0.3077 to 0.04436 | No | ns | 0.1885 |
| Mod vs. Mod+Anti | -0.2783 | -0.5718 to 0.01512 | No | ns | 0.0643 |
| Acu vs. Acu+Pro | 0.1817 | -0.0004078 to 0.3637 | No | ns | 0.0505 |
| Acu vs. Acu+Anti | 0.5983 | 0.4622 to 0.7344 | Yes | **** | <0.0001 |
| Day5 | | | | | |
| Con vs. Mod | 6.217 | 6.055 to 6.378 | Yes | **** | <0.0001 |
| Mod vs. Acu | -3.155 | -3.354 to -2.956 | Yes | **** | <0.0001 |
| Mod vs. Mod+Pro | -1.108 | -1.315 to -0.9018 | Yes | **** | <0.0001 |
| Mod vs. Mod+Anti | -0.5633 | -0.8141 to -0.3126 | Yes | *** | 0.0002 |
| Acu vs. Acu+Pro | -0.4683 | -0.7189 to -0.2178 | Yes | *** | 0.0008 |
| Acu vs. Acu+Anti | 0.7 | 0.3674 to 1.033 | Yes | *** | 0.0006 |
| Day7 | | | | | |
| Con vs. Mod | 8.577 | 8.345 to 8.809 | Yes | **** | <0.0001 |
| Mod vs. Acu | -3.635 | -3.863 to -3.407 | Yes | **** | <0.0001 |
| Mod vs. Mod+Pro | -0.7333 | -0.9732 to -0.4935 | Yes | **** | <0.0001 |
| Mod vs. Mod+Anti | -0.275 | -0.5194 to -0.03055 | Yes | * | 0.0257 |
| Acu vs. Acu+Pro | -1.217 | -1.391 to -1.042 | Yes | **** | <0.0001 |
| Acu vs. Acu+Anti | 0.2883 | 0.02916 to 0.5475 | Yes | * | 0.0297 |
| Day9 | | | | | |
| Con vs. Mod | 10.64 | 10.41 to 10.87 | Yes | **** | <0.0001 |
| Mod vs. Acu | -4.533 | -4.643 to -4.424 | Yes | **** | <0.0001 |
| Mod vs. Mod+Pro | -1.543 | -1.702 to -1.385 | Yes | **** | <0.0001 |
| Mod vs. Mod+Anti | -0.5067 | -0.6452 to -0.3682 | Yes | **** | <0.0001 |
| Acu vs. Acu+Pro | -1.608 | -1.749 to -1.468 | Yes | **** | <0.0001 |
| Acu vs. Acu+Anti | -0.06 | -0.4347 to 0.3147 | No | ns | 0.9891 |
