# Supplementary material for: Acupuncture Alleviates Neuroinflammation in Chronic Migraine by Modulating Lactobacillus and Its Metabolite Pathways
Source: Pain Res Manag. 2026 Jun 23;2026:5189419. doi: 10.1155/prm/5189419 (PMC13287961; doi:10.1155/prm/5189419)
Supplement: Supplementary file 13 — Supporting Information 13 Supporting Table S11: Statistical analysis of tail‐flick latency (seven‐group comparison). This table provides the statistical analysis of tail‐flick latency for the expanded seven‐group comparison, including descriptive statistics and between‐group comparisons. [file PRM-2026-5189419-s011.docx]

**Table S11** Statistical analysis of tail-flick latency（7 groups）.

| **Tukey's multiple comparisons test** | **Mean diff.** | **95.00% CI of diff.** | **Below threshold?** | **Summary** | **Adjusted *P* Value** |
| --- | --- | --- | --- | --- | --- |
| Day1 | | | | | |
| Mod vs. Acu | 0.01 | -0.2711 to 0.2911 | No | ns | >0.9999 |
| Mod vs. Mod+Pro | -0.02 | -0.3072 to 0.2672 | No | ns | >0.9999 |
| Mod vs. Mod+Anti | -0.02167 | -0.2926 to 0.2492 | No | ns | >0.9999 |
| Acu vs. Acu+Pro | -0.02833 | -0.2999 to 0.2432 | No | ns | 0.9996 |
| Acu vs. Acu+Anti | -0.01833 | -0.2732 to 0.2365 | No | ns | >0.9999 |
| Mod vs. Acu | 0.01 | -0.2711 to 0.2911 | No | ns | >0.9999 |
| Day3 | | | | | |
| Con vs. Mod | 0.8567 | 0.6911 to 1.022 | Yes | **** | <0.0001 |
| Mod vs. Acu | -0.4917 | -0.6644 to -0.3189 | Yes | **** | <0.0001 |
| Mod vs. Mod+Pro | -0.19 | -0.4279 to 0.04790 | No | ns | 0.1415 |
| Mod vs. Mod+Anti | -0.05167 | -0.2140 to 0.1107 | No | ns | 0.8717 |
| Acu vs. Acu+Pro | -0.04333 | -0.2334 to 0.1468 | No | ns | 0.9727 |
| Acu vs. Acu+Anti | 0.1683 | 0.004271 to 0.3324 | Yes | * | 0.0435 |
| Day5 | | | | | |
| Con vs. Mod | 1.193 | 0.9111 to 1.476 | Yes | **** | <0.0001 |
| Mod vs. Acu | -0.4567 | -0.7421 to -0.1712 | Yes | ** | 0.0025 |
| Mod vs. Mod+Pro | -0.3233 | -0.5994 to -0.04725 | Yes | * | 0.0212 |
| Mod vs. Mod+Anti | -0.1217 | -0.3935 to 0.1502 | No | ns | 0.6264 |
| Acu vs. Acu+Pro | -0.2267 | -0.4471 to -0.006206 | Yes | * | 0.043 |
| Acu vs. Acu+Anti | 0.08 | -0.1358 to 0.2958 | No | ns | 0.8064 |
| Day7 | | | | | |
| Con vs. Mod | 2.057 | 1.761 to 2.353 | Yes | **** | <0.0001 |
| Mod vs. Acu | -0.9533 | -1.266 to -0.6403 | Yes | **** | <0.0001 |
| Mod vs. Mod+Pro | -0.4367 | -0.7084 to -0.1649 | Yes | ** | 0.0055 |
| Mod vs. Mod+Anti | -0.07833 | -0.3599 to 0.2032 | No | ns | 0.9336 |
| Acu vs. Acu+Pro | -0.2317 | -0.5050 to 0.04171 | No | ns | 0.1103 |
| Acu vs. Acu+Anti | -0.04 | -0.3026 to 0.2226 | No | ns | 0.9936 |
| Day9 | | | | | |
| Con vs. Mod | 3.312 | 3.072 to 3.551 | Yes | **** | <0.0001 |
| Mod vs. Acu | -1.71 | -1.879 to -1.541 | Yes | **** | <0.0001 |
| Mod vs. Mod+Pro | -0.8517 | -1.044 to -0.6594 | Yes | **** | <0.0001 |
| Mod vs. Mod+Anti | -0.21 | -0.4514 to 0.03142 | No | ns | 0.0988 |
| Acu vs. Acu+Pro | -0.5167 | -0.6993 to -0.3341 | Yes | *** | 0.0001 |
| Acu vs. Acu+Anti | -0.03 | -0.1153 to 0.05528 | No | ns | 0.8313 |
